# The Effects of Different Modalities of an Acute Energy Deficit on Sleep and Next Morning Appetitive and Compensatory Behavior in Healthy Young Adults: The EDIES Protocol

**DOI:** 10.3390/nu15081962

**Published:** 2023-04-19

**Authors:** Oussama Saidi, Cyril Chatain, Giovanna C. Del Sordo, Rémi Demaria, Ludivine Lequin, Emmanuelle Rochette, Julie Larribaut, Mathieu Gruet, Pascale Duché

**Affiliations:** 1 Laboratory Impact of Physical Activity on Health (IAPS), Toulon University, F-83041 Toulon, France; 2 Psychology Department, New Mexico State University, 1780 E University Blvd, Las Cruces, NM 88003, USA; 3 Department of Pediatrics, Clermont-Ferrand University Hospital, F-63000 Clermont-Ferrand, France; 4 INSERM, CIC 1405, CRECHE Unit, Clermont Auvergne University, F-63000 Clermont-Ferrand, France

**Keywords:** sleep efficiency, energy balance, exercise, appetite, food reward, crossover design

## Abstract

Sleep is bi-directionally linked to energy balance. This crossover study design will evaluate the acute effect of a moderate energy deficit (500 kcal) induced by diet, exercise, or mixed (−250 kcal by diet and 250 kcal by exercise) on sleep and the next morning’s appetitive responses. The study sample comprises 24 healthy young adults. The experimental measurements will be conducted in a naturalistic, momentary manner and partly assessed by the participants. The participants will undergo a run-in period in order to stabilize their sleep schedules and provide them with training on the study protocol and measurements. Indirect calorimetry will be used to determine their resting metabolic rate and peak oxygen consumption (VO_2_ peak). Then, they will take part in a control session (CTL), followed by three energy deficit sessions in random order: a diet-induced energy deficit session (DED), an exercise-induced energy deficit session (EED), and a mixed energy deficit session (MED). All experimental sessions will be separated by a one-week washout. The participants’ sleep will be monitored by ambulatory polysomnography, and the next morning’s appetitive response will be evaluated via ad libitum food intake, appetite sensations, and food reward, measured by a food liking and wanting computerized test.

## 1. Introduction

### 1.1. Background and Rationale

A sustained negative energy balance (EB) is essential for weight loss to occur [1,2,3]. While both exercise and dietary restrictions can be used to induce an energy deficit, they do not seem to induce the same adaptive responses for appetite [4]. It has been shown that an energy deficit induced by dietary restrictions alone promotes compensatory appetitive responses that counter-regulate negative EBs by increasing following energy intake [5]. Interestingly, an isocaloric energy deficit (ED) induced by exercise was found to limit this compensatory response [6]. 

Recent studies have shown that altered sleep patterns while undertaking weight loss programs condition the adherence to and the success of the latter [7,8]. More importantly, it appears that sleep disruption affects the control of EB, mainly through an upregulation (≈250 kcal) of energy intake via various pathways, including appetite hormones, cognitive control, and reward mechanisms [9,10]. Furthermore, it was also found that sleep curtailment during caloric restriction undermines fat loss in favor of increased fat-free mass loss [11,12].

Studies carried out on animal models show that a prolonged restriction of food intake in rats induces a deterioration of sleep, marked by an increase in the time spent awake and a shortening of slow-wave sleep [13,14]. In humans, there is a paucity of evidence documenting the effect of moderate caloric restriction (similar to that induced in clinical practice for weight loss purposes) on sleep. However, some studies underlined that large energy restrictions [≈300 kcal/d] and prolonged fasting lead to a drastic drop in nocturnal melatonin secretion (−20%) [15,16,17]. Melatonin is a hormone produced by the pineal gland in the brain in response to darkness and plays a key role in sleep. Indeed, by lowering the core body temperature, increased melatonin secretion during the evening reduces arousal and increases sleep propensity [18]. Thus, a melatonin deficiency has been related to insomnia and sleep disturbances [19]. Furthermore, Karklin et al. (1994) showed during a dietary weight loss intervention in overweight women with a caloric intake of 800 kcal/d, an alteration of sleep marked by an increase in sleep latency and a decrease in the time spent in slow-wave sleep (SWS) [20]. Moreover, observations of bodybuilders during the “cutting” phase of contest preparation also reported poor sleep outcomes. These periods were generally characterized by severe dietary energy restriction [21,22]. While dietary restrictions may alter sleep, exercise seems to have a completely converse effect. This has been extensively studied over the years, revealing an increase in total sleep time, sleep efficiency (SE), slow-wave sleep (SWS), and rapid eye movement sleep (REM), as well as a decrease in wake after sleep onset in response to acute and chronic exercise [23,24]. However, whether or not these effects are maintained during a negative EB state needs to be determined to draw firm conclusions.

### 1.2. Aims

The main objective of this study is to evaluate the acute effect of a moderate energy deficit (500 kcal) induced by diet, exercise, or both (mixed: −250 kcal by diet and 250 kcal by exercise) on the quality and architecture of sleep in healthy young adults. The secondary objectives will be to investigate the effect of the energy deficit modality on (i) next morning appetitive responses ([i_a_] ad libitum food intake, [i_b_] appetite sensations, [i_c_] food reward, (ii) sleepiness, and (iii) fatigue.

## 2. Materials and Methods

### 2.1. Study Setting and Design

The “Exercise and/or Diet induced Iso-Energetic deficit effect on Sleep” (EDIES) is a single-center study with a crossover design in which each participant will be allocated to a randomized sequence of experimental sessions and, hence, acts as his own control. This trial will be conducted in Toulon, France (Toulon University, laboratory impact of physical activity on health [IAPS]), and received approval from an Institutional Review Board (IRB00012476-2023-28-02-235) with all undertaken assessments complying with the principles of the Declaration of Helsinki. The measurements are realized in a naturalistic, momentary manner and partly assessed by the participants in an ecological setting. The inclusion will be launched in March 2023, and a sufficient number of participants is expected to be reached within three months following the first inclusion.

**Figure 1:**
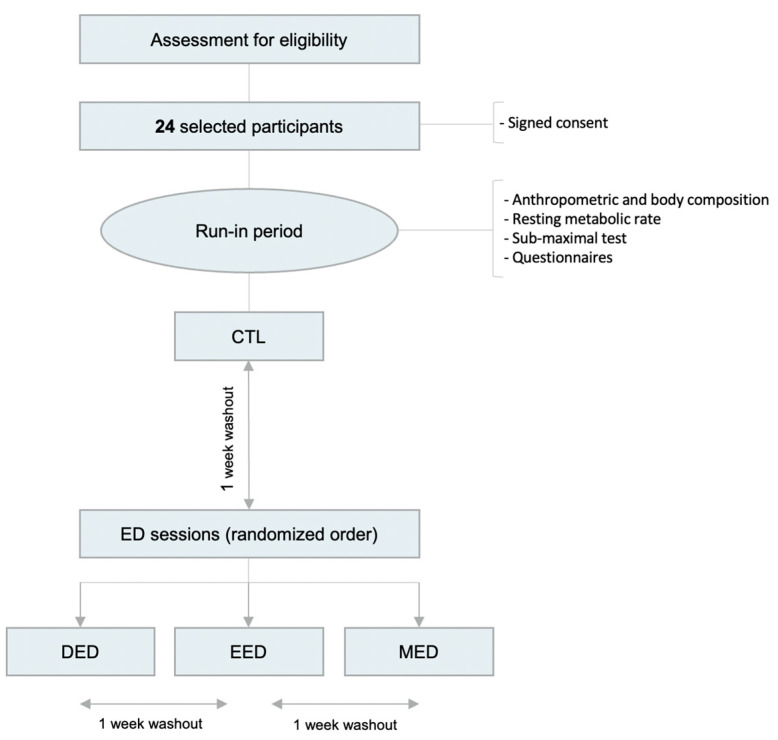
Flow diagram of the EDIES study: CTL, control session; DED, diet-induced energy deficit session; ED, energy deficit; EED, exercise-induced energy deficit session; MED, mixed energy deficit session.

Upon reception of the signed consent, the participants will undergo a run-in week. This will be followed by a control session (CTL), after which energy deficit sessions (diet-induced energy deficit session [DED], exercise-induced energy deficit session [EED], and mixed energy deficit session [MED]) will take place in random order. All experimental sessions (including the CTL) will be held for 28 h and separated by a one-week washout period (Figure 1).

### 2.2. Participants

#### 2.2.1. Sample Size

An a priori analysis was conducted to establish the number of subjects needed to obtain a statistical power of 80%. Based on a repeated-measures design (four within the measures) for our primary outcome of interest (SE), it was found that 24 subjects would be required for the following parameters: alpha = 0.05, effect size f = 0.25 (medium effect), correlation among repeated measures = 0.5, and sphericity correction = 1. 

#### 2.2.2. Recruitment

The participants will be recruited from a pool of college students through flyers and university e-mail. Then, interested individuals will first be reached by video conferencing to conduct a screening and brief them on the experimental protocol of the study and measurements. Afterward, selected individuals will be invited to a laboratory meeting and informed of all research protocols and details. In light of the information sheet, the first author (O.S.) will request written consent from those willing to take part in the study.

#### 2.2.3. Eligibility Criteria

Participants meeting the following criteria will be eligible for the study: (1) be a male; (2) be aged between 19 and 24 years (inclusive); (3) be normal-weighted (BMI: 18.5 to 25 kg/m^2^); (4) have a moderate level of physical activity (i.e., no more than 3 intense exercise sessions per week); and (5) be able to read and understand the French language. 

The non-inclusion criteria are as follows: (1) being involved in another research protocol during the inclusion period; (2) having a specific diet; and (3) having a medical history and medication not compatible with the study (e.g., corticosteroids, sleeping pills), or any other chronic illnesses or injuries that may interfere with the subject’s ability to perform the exercises or that modifies their sleep and eating behaviors; and (4) shift work/severe jet lag.

All diagnosed sleep disorders and/or pathologies will be considered exclusion criteria. All recruited participants will be screened for obstructive sleep apnea syndrome (OSA) using the Berlin questionnaire [25] and the polysomnographic recording of the habituation night (i.e., apnea/hypopnea index > 5 events/hour).

### 2.3. Randomization

#### 2.3.1. Sequence Generation

Upon reception of the signed consent, the participants will be subjected to a random allocation through a computer-generated list (created with “randomization.com”) (Kim and Shin, 2014). According to our within-subjects design, random permutations of interventions will be computed. Thus, each subject can be allocated to a specific order of interventions (any combination of EED, MED, or DED). The sequence allocation will be generated by the study’s statistician and provided to the investigators conducting the assessments. This document will be securely stored and password-protected on the University of Toulon’s online servers and only shared with the team members directly engaged in the study’s protocol.

#### 2.3.2. Concealment and Blinding

Given the nature of the study, concealing allocated interventions to subjects or investigators is not feasible. The subjects will go through all interventions sequentially and will explicitly know what is expected of them in each intervention. Therefore, the subjects and investigators will not be blinded to the interventions’ allocation. However, the investigators will be blinded to the subjects’ allocation until the end of the baseline assessments as well as randomization assignments during the data analysis process (e.g., sleep analysis).

### 2.4. Run-In Period and Baseline Assessments

#### 2.4.1. Run-In Period Overview

The run-in period will take place one week before the launch of the experimental sessions in order to accomplish the following goals: (1) provide detailed information on the study flow to each participant; (2) stabilize the sleep window from 9:45 p.m. to 6:45 a.m. (compliance will be verified using accelerometers [Table S1]); (3) provide training for the participants on the use of the study’s measuring instruments (e.g., wearing the ambulatory polysomnographic device [PSG]); (4) complete baseline assessments; and (5) familiarize the participants to sleep with the PSG device.

#### 2.4.2. Baseline Assessments

- Anthropometric and body composition: Body mass will be measured using a digital weight scale (UM-076, TANITA, Tokyo, Japan). Height will be assessed using a portable stadiometer with the participants barefooted (HR001, TANITA, Tokyo, Japan). The skinfold thickness will be measured in duplicate by the same investigator on the right side of the body at the biceps, triceps, subscapular, and supra-iliac sites using a Harpenden caliper (Baty International, Burgess Hill, UK). Relative fat mass will be then calculated using Siri equations amended by Weststrate and Deurenberg (1989) [26].
- The resting metabolic rate (RMR) and submaximal test: The resting metabolic rate will be measured in the morning, under a fasted state, by indirect calorimetry using a mobile spiroergometric system (METAMAX 3B-R2, CORTEX Biophysik GmbH, Leipzig, Germany). Before each test, the equipment will be calibrated according to the manufacturer’s recommendations. The participants will be placed in a supine position in a thermoneutral environment (22–25 °C room temperature) for 45 min before starting the measurements. After reaching a steady state, the O_2_ consumption and CO_2_ production, normalized for temperature, barometric pressure, and humidity, will be recorded and averaged at one-minute intervals for 20–45 min and averaged over the entire measurement period. The resting energy expenditure (in kcal/day) and the respiratory quotient (CO_2_/O_2_ ratio) will be calculated thereafter. The resting metabolic rate assessment will be followed by a submaximal test in order to estimate the peak oxygen consumption (VO_2_ peak) and therefore calibrate the energy deficit sessions’ exercise. The exercise will be performed on an adjustable cycle ergometer (Wattbike Ltd., Nottingham, UK). After a warm-up period (2 min), performed at 45 W, the output will be increased by 15 W every 5 min (allowing to ensure a stable state for each step) until the participants reach 60% of the age-predicted maximum heart rate amended by Tanaka et al. (2001) [27]. The heart rate (HR) will be continuously recorded using a heart rate sensor (Polar H10, Polar Electro, Kempele, Finland). The energy expenditure (EE) will be estimated for each step by multiplying the O_2_ consumption (VO_2_) by the energy equivalent (kcal/L O_2_) for each participant. The five steps will allow for the establishment of the HR–VO_2_ relationship for each participant, which will be used to calibrate the exercise intensity required for an expenditure of 125 and 250 kcal.
- Questionnaires: The to-be-completed questionnaires will include (1) the International Physical Activity Questionnaire (IPAQ) to estimate the physical activity level [28]; (2) the Morningness-Eveningness Questionnaire (MEQ) to determine the participants’ chronotype (i.e., evening, intermediate, or morning typology) [29]; (3) the Pittsburg Sleep Quality Index (PSQI) to assess the quality of sleep over the last month [30]; (4) the Dutch Eating Behavior Questionnaire (DEBQ) with scales for restrained, emotional and external eating behaviors [31]; and (5) the Multidimensional Fatigue Inventory (MFI) to assess fatigue traits [32].

### 2.5. Experimental Sessions

#### 2.5.1. Control Session

**Figure 2:**
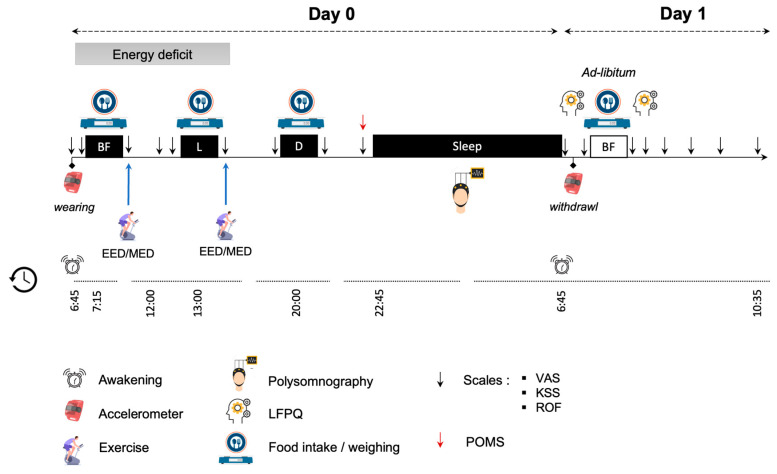
Experimental session design: BF, breakfast; D, dinner; EED, exercise-induced energy deficit session; KSS, Karolinska Sleepiness Scale; L, lunch; LFPQ, Leeds Food Preference Questionnaire; MED, mixed energy deficit session; POMS, Profile of Mood States questionnaire; ROF, Rating-of-Fatigue Scale; VAS, visual analogue scale.

The control session (CTL) will be carried out over 28 h and starts when the participant wakes up (6:45 a.m.). Upon waking up, the participants will be equipped with accelerometers (GT3X+, ActiGraph LLC, Pensacola, FL, USA) on their waists until the next morning to monitor their physical activity. They will be asked to maintain their habitual overall level of physical activity, and any structured physical exercise during and 48 h before the session will be prohibited. Food intake will be organized over three scheduled meals (breakfast (7:15), lunch (12:30), and dinner (20:00)). The meals offered will be adapted to each participant’s preferences based on a panel of dishes proposed by the investigators (Table S2). The participants will be asked to take pictures of every consumed meal (habitual meal) and to fill out a food diary by weighing each consumed food item using a kitchen scale (EBC9i, EssentielB, Fretin, France) provided by the investigators. Appetite, sleepiness, and fatigue will be assessed throughout the day (i.e., 12 measures; Figure 2) using the Karolinska Sleepiness Scale (KSS) [33], Rating-of-Fatigue Scale (ROF) [34] and appetite visual analogue scale (VAS; 150 mm) [35], respectively. The Profile of Mood States questionnaire (POMS) [36] will be completed before bedtime. During the night, sleep will be measured by ambulatory polysomnography. The next morning, the participants will be asked to return to the laboratory in a fasting state. They will complete the Leeds Food Preference Questionnaire (LFPQ) [37,38] on a computer before being served a standardized ad libitum meal (buffet-style meal). After the meal, they will complete the LFPQ a second time. Sleepiness, fatigue, and appetite will be assessed throughout the morning (i.e., 8 measures; Figure 1). The control session will end at 10:35. 

The data collected during the control session will be used to assess the energy balance of each subject and to calibrate the food intake and exercises of the energy deficit sessions.

#### 2.5.2. Energy Deficit Sessions

All experimental sessions will be conducted similarly to the CTL session. As explained above, the sessions’ order will be randomized and counterbalanced. All meals will be taken at the same time, similar to the CTL session. All measurements will also be organized in the same way, and the participants will be asked to maintain the same level of physical activity as in the CTL session during the 3 experimental sessions, with no structured exercise during and 48 h before the start of each session. Physical activity will again be monitored by accelerometry. Thus, the only difference will be the energy deficit of 500 kcal, induced by the energy balance assessed during the CTL session. Energy deficits will be induced by 3 modalities corresponding to the 3 energy deficit sessions:

- DED session: 500 kcal dietary deficit (−250 kcal on breakfast and −250 kcal on lunch);
- EED session: 500 kcal deficit, induced by two exercise bouts (−250 kcal after breakfast and −250 kcal after lunch);
- MED session: 250 kcal dietary deficit (−125 kcal at breakfast and −125 kcal at lunch) and a 250 kcal deficit induced by exercise (−125 kcal after breakfast and −125 kcal after lunch).

During the DED and MED sessions, the food intakes will be replicated identically to the CTL session, while the portion sizes will be adapted to induce the desired energy deficit (i.e., the same standard food items with reduced amounts). During the EED and MED sessions, two exercise bouts will be performed on a cycle ergometer at the target HR corresponding to 75% VO_2peak_ at the same time (after breakfast and lunch, respectively). The only difference between the EED and MED sessions is a modification of the exercise intensity to induce the desired energy expenditure.

### 2.6. Assessement of Energy Balance 

The energy balance will be calculated individually for each participant during each experimental session. The EI will be calculated based on the computerized nutrient analysis software (Bilnut 4.0 SCDA, Nutrisoft) and the Ciqual tables (2020 version) based on the weight and food items recorded by the participants during Day 0 of each session. Energy expenditure (EE) will be calculated based on the RMR, daily metabolic equivalent of the task values derived from the accelerometer’s raw data for daily physical activity [39], and exercise energy expenditure (during EED and MED sessions).

### 2.7. Outcomes

#### 2.7.1. Primary Outcome: Sleep Efficiency

SE is defined as the ratio between total sleep time (TST) and total time spent in bed (TBT). It is given as a percentage. SE will be measured objectively during each session (i.e., CTL, DED, EED, MED) using an ambulatory polysomnograph (Sleep Profiler-PSG2, Advances Brain Monitoring, Carlsbad, CA, USA). The device is a portable multi-channel recorder that allows the acquisition of the following: the electroencephalographic (EEG), electrooculographic (EOG) and electromyographic (EMG) signals from three frontopolar channels; the airflow through a nasal cannula and pressure transducer; head movements and position by actigraphy; snoring, with an acoustic microphone; the pulse, using a forehead and finger sensor; the oximetry, using a wireless wrist oximeter; and the thorax and abdomen efforts by respiratory inductive plethysmography. The records will then be extracted through the Sleep Profiler portal. Validated auto-staging will be applied based on the ratios of the power spectral densities and the auto-detection of cortical and micro-arousals, sleep spindles, and ocular activity [40,41,42], which will be reviewed and modified by a sleep expert if needed. 

#### 2.7.2. Experimental Secondary Outcomes

- Other sleep outcomes: Beyond SE, the Sleep Profiler-PSG2 provides other sleep variables, such as the following: total sleep time (TST); sleep onset latency (SOL); wake after sleep onset (WASO); the number of awakenings lasting more than 30 s; the arousal index and staging (time spent in non-rapid (NREM; Stage-1, Stage-2, and Stage-3) and rapid eye movement (REM)), according to the American Academy of Sleep Medicine recommendations [43].
- Mood: The Profile of Mood States will be used to assess the participant’s mood before the night of each experimental session [36]. All participants will be asked to rate “How are you feeling right now?” using 24 mood descriptors (e.g., nervous, unhappy, etc.). For each descriptor, the participants have to answer using a 5-point Likert scale, from 0 (not at all) to 4 (extremely). This questionnaire is divided into six subscales (fatigue, confusion, vigor, depression, tension, and anger), each containing four mood descriptors.
- Ad libitum energy intake: A standardized buffet breakfast will be organized on day1 of each session [44]. Consumed food items will be weighed and recorded by the investigators. Subsequently, the computerized nutrient analysis software (Bilnut 4.0 SCDA, Nutrisoft) and Ciqual tables (2020 version) will calculate the energy intake and the part of energy derived from each class of macronutrients.
- Food liking and wanting: LFPQ will be used to assess the individual’s food preferences [37]. It comprises two sub-tasks that require interactions from the participant. The first task (explicit task) involves an explicit assessment of food pictures using the 100-unit VAS. Single food images are randomly displayed to the participant on a screen computer, who is required to rate it according to “How pleasant would it be to taste some of this food now?” (explicit liking) and “How much do you want some of this food now?” (explicit wanting). The second task (implicit or forced choice task) requires a quick choice to be made between paired combinations of food pictures from different categories. During this task, a series of food image pairs are presented to the participant with the instruction, “Which food do you most want to eat now?”.
- Appetite sensations: Sensations of hunger, appetite, and a desire to eat will be measured using a VAS (150 mm) throughout all sessions (i.e., 9 measures on day 0, and 8 measures on day 1). These VASs were previously validated by Flint et al. (2000) [35].
- Sleepiness: The subjective level of sleepiness will be measured using the KSS [33]. The participant has to rate his subjective sensation of sleepiness in the last 10 min using a 9-point scale, ranging from 1 (extremely alert) to 9 (extremely sleepy).
- Fatigue: The French-validated version of the ROF scale will be used to assess the state of fatigue [34]. This is an 11-point scale, from 0 (not fatigued at all) to 10 (total fatigue and exhaustion), with accompanying descriptors and schematic components, which allow for tracking perceived fatigue across different ranges of daily life, physical activity, and recovery contexts.

#### 2.7.3. Baseline Secondary Outcomes

- Subjective sleep quality: The PSQI questionnaire will be used to assess sleep quality [30]. It comprises 19 self-reported questions and measures seven components (i.e., overall sleep quality, sleep latency, sleep duration, sleep efficiency, sleep disturbances, use of medication for sleep, and daytime dysfunction due to sleepiness). The sum of scores from the seven components provides a global score from 0 (better) to 21 (worse). A global score of ≤5 is associated with good sleep quality; in reverse, a global score of ≥5 is associated with poor sleep quality.
- Eating behaviors: Eating behaviors will be measured using the DEBQ, a 33-item questionnaire assessing three distinct eating behaviors: emotional eating (13 items), external eating (10 items), and restrained eating (10 items) [31]. For each item, the participants have to answer using a 5-point Likert scale, from 1 (never) to 5 (very often), with higher scores indicating greater endorsement of the eating behavior.
- Chronotype: The MEQ will be used to define each participant’s chronotype [29]. The participants have to score 19 items using a 5-point Likert scale. The sum of the item scores ranges from 16 to 86. Scores of 41 and below indicate “evening types”, scores of 59 and above indicate “morning types”, and scores between 42–58 indicate “intermediate types”.
- Subjective physical activity. The short form (7 questions) of IPAQ will be used to assess the participant’s physical activity level [28]. The participants will be asked to report the time spent being physically active in the last 7 days using four different dimensions (i.e., vigorous physical activity, moderate physical activity, time to walk, and time spent sitting). Data collected with this questionnaire allow the classification of participants into three categories: inactive, minimally active, health-enhancing physical activity and/or to obtain a continuous measure reported as MET-minutes for each dimension (walking MET-min/week, moderate MET-min/week, and vigorous MET-min/week).
- Fatigue trait. The fatigue trait will be measured using the MFI questionnaire [32], which includes 20 items addressing different dimensions of fatigue, divided into 5 categories: general fatigue, physical fatigue, mental fatigue, reduced activities, and reduced motivation. The questionnaire includes positively and negatively worded items that are rated using a 5-point Likert scale. The dimension subscores (4–20) and global scores (20–100) can be calculated, with a high score indicating a high degree of fatigue.

### 2.8. Strategies to Improve Study Adherence and Compliance

Several strategies will be established to maximize adherence and compliance with the study protocol. A booklet containing the food notebook and the questionnaires to be completed throughout the study will be provided to each eligible participant. This booklet will highlight the specific timings at which participants should fill out the questionnaires and record their food intake in an easy and convenient manner. Participants will be able to choose between three food menus, depending on their preferences. They will be provided with a box of easy-to-cook food items and a kitchen scale to facilitate meal preparation and the recording of food intake at their own home. In order to reinforce engagement in the study, the participants will receive reminder messages from the investigators. They will also have the possibility to communicate with the investigators through phone calls throughout the duration of the study. If necessary, they will be encouraged to ask questions and reminded of specific protocol instructions.

### 2.9. Data Management and Confidentiality

Each participant will be given a unique numerical/letter identifier, which will be used for all assessment documents. Data will be collected via paper and electronic database formats, in which only the participants’ unique identifiers will be used. Informed consent, with participants’ names, will be kept separately from the other documents in a locked filing cabinet by the principal investigator. A list of the participants’ names and their unique identifiers will be kept in a password-protected file, which will only be accessible to the research team involved in this project. After a period of 5 years, all documents mentioning the participants’ names will be shredded and disposed of safely. 

### 2.10. Statistical Methods

Statistical analysis and graphics will be performed using R Studio (version 4.0.5, RCore Team, 2021) and Prism 9 (GraphPad, San Diego, CA, USA). Continuous variables will be presented as means and standard deviations, subject to the normality of their distributions (using a Shapiro–Wilk test if necessary). In case of non-normality, they will be presented as medians, quartiles, and extreme values. Data manipulation, through logarithmic transformation, will only happen if violations of assumptions for each test are found. Non-parametric testing will be used if necessary: Friedman tests and Wilcoxon signed-rank tests for post hocs will be computed instead of one-way repeated ANOVAs; the Wilcoxon tests will replace the paired samples *t*-tests. As far as possible, graphic representations will be associated with these analyses.

#### 2.10.1. Statistical Analysis for Sleep Outcomes

For the sleep outcomes (TST, SOL, SE, WASO, REM, N1, N2, N3, and arousals), the differences between interventions (EED, MED, and DED) will be assessed using a repeated-measures ANOVA by comparing the three interventions as a within-subject factor. If significance is obtained, post hoc pairwise comparison tests with a Tukey HSD correction will be applied. Sleep measures during the interventions will also be compared to the baseline to evaluate the effect of each intervention separately, using paired samples *t*-tests. The effect sizes will be computed to assess the strength of the relationships: Cohen’s d for the *t*-tests and eta^2^ for the main effects.

#### 2.10.2. Statistical Analysis for Secondary Outcomes

The POMS questionnaire (taken before bedtime in each intervention) will be analyzed using repeated-measures ANOVA to assess the differences between interventions (followed by the Tukey HSD post hoc tests if necessary) and paired samples *t*-tests to evaluate the effect of each intervention separately against the baseline measures. 

The LFPQ will be administered twice, before and after an ad libitum meal on day 2. The paired samples *t*-tests will be computed for each intervention separately to assess the pre-post differences and the differences between the baseline and intervention measurements. A repeated-measures ANOVA will also be computed on the delta (post-pre LFPQ scores) to compare the score differences between interventions. Tukey’s HSD post hoc tests will be performed if significance is reached. Similarly, the food intake during the ad libitum meal will be analyzed using repeated-measures ANOVA (interventions comparisons) and the paired samples *t*-tests (the baseline-intervention comparisons). 

For the Karolinska, ROF, and hunger sensation scale, several measures throughout the day will be taken. In order to assess the time course of these measures for each day, the area under the curve (AUC) for each questionnaire will be calculated using the trapezoid method and analyzed using repeated-measures ANOVA for the differences between the interventions and using the paired samples *t*-tests for the baseline-intervention comparisons.

Manipulation checks will be performed (paired samples *t*-tests) on the energy balance measure between each energy deficit session to ensure that the induced energy deficit was isocaloric.

#### 2.10.3. Plan to Handle Missing Data

Missing data will be handled, as outlined in Jacobsen et al. (2017) [45]. Data will be analyzed as a complete case analysis if less than 5% of the data points are missing. If more than 5% of the data is missing in the entire sample, multiple imputations techniques will be performed and then followed by sensitivity analyses [45].

#### 2.10.4. Methods for Additional Statistical Analyses

We do not preclude the conduction of additional statistical analyses on the sample’s data. These have not been planned yet and will be reported as non-planned analyses in future reports.

#### 2.10.5. Plan to Give Access to Full Protocol, Data, and Statistical Code

All datasets and statistical codes used in this trial will be made available, upon reasonable request, by the corresponding author. Any shared data will not include the participants’ identifiers. 

## 3. Discussion and Future Perspectives

From an integrative point of view, the literature in physiology argues that there is an interaction between the energy state and sleep in mammalians. It was suggested that during periods of negative energy balance, neuronal populations, being part of the sleep-wake and metabolic pathways, favor arousal instead of sleep for foraging and survival ends [46].

Given that sleep disruption increases energy intake, appetite, and food rewards [47,48], the elaboration of appropriate interventions intended to promote a sustainable negative energy balance while avoiding compensatory behaviors require the establishment of good sleep quality. On the one hand, this study will allow a better understanding of the interaction between the different modalities of energy deficit and sleep. On the other hand, it will provide additional information on the role of sleep in the following compensatory eating behaviors, with an emphasis on appetite sensations and food reward.

Hunger sensations were implicated in the difficulties initiating and maintaining sleep [49]. Previous studies showed that ED, induced by dietary restriction, potentiates these sensations more than exercise ED [4,6]. Therefore, we hypothesize that exercise ED might be more effective in improving the quality of sleep while reducing appetitive compensatory behaviors in the following morning. In previous work, we showed that an intensive single bout of exercise inducing an ED of almost 500 kcal among adolescent girls with obesity succeeded in improving sleep efficiency (+5%) while reducing next-morning energy-dense food consumption [50]. However, this study was limited by the accelerometry-based measure of sleep and the absence of a detailed assessment of the appetitive response. In addition to addressing these shortcomings, the EDIES study will also allow the evaluation of the feasibility of including this pilot protocol in the clinic. 

The effect of moderate ED on sleep is understudied in the current literature. Therefore, the results of this study will be valuable. Including females requires control for their menstrual cycles, as it directly affects sleep and other physiological regulatory processes that may impact their eating behavior, which would considerably extend the study duration [51]. However, the perspective will be to be more inclusive in testing this protocol among different populations, including the female sex, young populations (children and adolescents), and, more importantly, participants with obesity, especially as obesity might play a role in modifying the adaptations that will be observed in healthy participants. Moreover, we tested the effect of an early ED. Future studies comparing early vs. late ED impact on sleep would be very interesting. Finally, the medium-to-long-term adaptation of sleep to ED and weight loss remains to be determined and should be addressed in future longitudinal designs.

## Supplementary Materials

The following supporting information can be downloaded at: https://www.mdpi.com/article/10.3390/nu15081962/s1, Table S1: Accelerometry analyses, Table S2: Food items included in the ad libitum buffet meals and the standardized meals.

## References

[1] Aragon A.A., Schoenfeld B.J., Wildman R., Kleiner S., VanDusseldorp T., Taylor L., Earnest C.P., Arciero P.J., Wilborn C., Kalman D.S. (2017). International Society of Sports Nutrition Position Stand: Diets and Body Composition. J. Int. Soc. Sports Nutr..

[2] Hall K.D. (2008). What Is the Required Energy Deficit per Unit Weight Loss?. Int. J. Obes..

[3] Strasser B., Spreitzer A., Haber P. (2007). Fat Loss Depends on Energy Deficit Only, Independently of the Method for Weight Loss. Ann. Nutr. Metab..

[4] Thivel D., Metz L., Julian V., Isacco L., Verney J., Ennequin G., Charlot K., Beaulieu K., Finlayson G., King J.A. (2021). Diet-but Not Exercise-Induced Iso-Energetic Deficit Induces Compensatory Appetitive Responses. Eur. J. Clin. Nutr..

[5] O’Connor K.L., Scisco J.L., Smith T.J., Young A.J., Montain S.J., Price L.L., Lieberman H.R., Karl J.P. (2016). Altered Appetite-Mediating Hormone Concentrations Precede Compensatory Overeating after Severe, Short-Term Energy Deprivation in Healthy Adults. J. Nutr..

[6] Thivel D., Finlayson G., Miguet M., Pereira B., Duclos M., Boirie Y., Doucet E., Blundell J.E., Metz L. (2018). Energy Depletion by 24-h Fast Leads to Compensatory Appetite Responses Compared with Matched Energy Depletion by Exercise in Healthy Young Males. Br. J. Nutr..

[7] Ross K.M., Graham Thomas J., Wing R.R. (2016). Successful Weight Loss Maintenance Associated with Morning Chronotype and Better Sleep Quality. J. Behav. Med..

[8] Thomson C.A., Morrow K.L., Flatt S.W., Wertheim B.C., Perfect M.M., Ravia J.J., Sherwood N.E., Karanja N., Rock C.L. (2012). Relationship between Sleep Quality and Quantity and Weight Loss in Women Participating in a Weight-loss Intervention Trial. Obesity.

[9] Chaput J.-P., McHill A.W., Cox R.C., Broussard J.L., Dutil C., da Costa B.G., Sampasa-Kanyinga H., Wright K.P. (2023). The Role of Insufficient Sleep and Circadian Misalignment in Obesity. Nat. Rev. Endocrinol..

[10] St-Onge M.-P. (2013). The Role of Sleep Duration in the Regulation of Energy Balance: Effects on Energy Intakes and Expenditure. J. Clin. Sleep Med..

[11] Nedeltcheva A.V., Kilkus J.M., Imperial J., Schoeller D.A., Penev P.D. (2010). Insufficient Sleep Undermines Dietary Efforts to Reduce Adiposity. Ann. Intern. Med..

[12] Stich F.M., Huwiler S., D’Hulst G., Lustenberger C. (2022). The Potential Role of Sleep in Promoting a Healthy Body Composition: Underlying Mechanisms Determining Muscle, Fat, and Bone Mass and Their Association with Sleep. Neuroendocrinology.

[13] Alvarenga T.A.F., Andersen M.L., Papale L.A., Antunes I.B., Tufik S. (2005). Influence of Long-Term Food Restriction on Sleep Pattern in Male Rats. Brain Res..

[14] Dewasmes G., Duchamp C., Minaire Y. (1989). Sleep Changes in Fasting Rats. Physiol. Behav..

[15] Michalsen A., Schlegel F., Rodenbeck A., Lüdtke R., Huether G., Teschler H., Dobos G.J. (2003). Effects of Short-Term Modified Fasting on Sleep Patterns and Daytime Vigilance in Non-Obese Subjects: Results of a Pilot Study. Ann. Nutr. Metab..

[16] Peuhkuri K., Sihvola N., Korpela R. (2012). Dietary Factors and Fluctuating Levels of Melatonin. Food Nutr. Res..

[17] Röjdmark S., Wetterberg L. (1989). Short-term Fasting Inhibits the Nocturnal Melatonin Secretion in Healthy Man. Clin. Endocrinol..

[18] Zhdanova I.V., Lynch H.J., Wurtman R.J. (1997). Melatonin: A Sleep-Promoting Hormone. Sleep.

[19] Zisapel N. (2007). Sleep and Sleep Disturbances: Biological Basis and Clinical Implications. Cell. Mol. Life Sci..

[20] Karklin A., Driver H.S., Buffenstein R. (1994). Restricted Energy Intake Affects Nocturnal Body Temperature and Sleep Patterns. Am. J. Clin. Nutr..

[21] Fagerberg P. (2018). Negative Consequences of Low Energy Availability in Natural Male Bodybuilding: A Review. Int. J. Sport Nutr. Exerc. Metab..

[22] Pardue A., Trexler E.T., Sprod L.K. (2017). Case Study: Unfavorable but Transient Physiological Changes during Contest Preparation in a Drug-Free Male Bodybuilder. Int. J. Sport Nutr. Exerc. Metab..

[23] Kredlow M.A., Capozzoli M.C., Hearon B.A., Calkins A.W., Otto M.W. (2015). The Effects of Physical Activity on Sleep: A Meta-Analytic Review. J. Behav. Med..

[24] Driver H.S., Taylor S.R. (2000). Exercise and Sleep. Sleep Med. Rev..

[25] Netzer N.C., Stoohs R.A., Netzer C.M., Clark K., Strohl K.P. (1999). Using the Berlin Questionnaire to Identify Patients at Risk for the Sleep Apnea Syndrome. Ann. Intern. Med..

[26] Weststrate J.A., Deurenberg P. (1989). Body Composition in Children: Proposal for a Method for Calculating Body Fat Percentage from Total Body Density or Skinfold-Thickness Measurements. Am. J. Clin. Nutr..

[27] Tanaka H., Monahan K.D., Seals D.R. (2001). Age-Predicted Maximal Heart Rate Revisited. J. Am. Coll. Cardiol..

[28] Lee P.H., Macfarlane D.J., Lam T., Stewart S.M. (2011). Validity of the International Physical Activity Questionnaire Short Form (IPAQ-SF): A Systematic Review. Int. J. Behav. Nutr. Phys. Act..

[29] Taillard J., Philip P., Chastang J.-F., Bioulac B. (2004). Validation of Horne and Ostberg Morningness-Eveningness Questionnaire in a Middle-Aged Population of French Workers. J. Biol. Rhythm..

[30] Buysse D.J., Reynolds C.F., Monk T.H., Berman S.R., Kupfer D.J. (1989). The Pittsburgh Sleep Quality Index: A New Instrument for Psychiatric Practice and Research. Psychiatry Res..

[31] Van Strien T., Frijters J.E., Bergers G.P., Defares P.B. (1986). The Dutch Eating Behavior Questionnaire (DEBQ) for Assessment of Restrained, Emotional, and External Eating Behavior. Int. J. Eat. Disord..

[32] Gentile S., Delarozière J.C., Favre F., Sambuc R., San Marco J.L. (2003). Validation of the French ‘Multidimensional Fatigue Inventory’(MFI 20). Eur. J. Cancer Care.

[33] Åkerstedt T., Anund A., Axelsson J., Kecklund G. (2014). Subjective Sleepiness Is a Sensitive Indicator of Insufficient Sleep and Impaired Waking Function. J. Sleep Res..

[34] Brownstein C.G., Rimaud D., Singh B., Fruleux-Santos L.-A., Sorg M., Micklewright D., Millet G.Y. (2021). French Translation and Validation of the Rating-of-Fatigue Scale. Sports Med. Open.

[35] Flint A., Raben A., Blundell J.E., Astrup A. (2000). Reproducibility, Power and Validity of Visual Analogue Scales in Assessment of Appetite Sensations in Single Test Meal Studies. Int. J. Obes..

[36] Cayrou S., Dickès P., Dolbeault S. (2003). Version Française Du Profile of Mood States (POMS-f). J. Thérapie Comport. Cogn..

[37] Finlayson G., King N., Blundell J.E. (2007). Liking vs. Wanting Food: Importance for Human Appetite Control and Weight Regulation. Neurosci. Biobehav. Rev..

[38] Leenaars C.H., Zant J.C., Aussems A., Faatz V., Snackers D., Kalsbeek A. (2016). The Leeds Food Preference Questionnaire after Mild Sleep Restriction—A Small Feasibility Study. Physiol. Behav..

[39] Crouter S.E., Churilla J.R., Bassett D.R. (2008). Accuracy of the Actiheart for the Assessment of Energy Expenditure in Adults. Eur. J. Clin. Nutr..

[40] Westbrook P.R., Levendowski D.J., Zavora T., Davis G., Popovic D., Berka C., Mitrovic M., Veljkovic B. (2014). System for the Assessment of Sleep Quality in Adults and Children. U.S. Patent.

[41] Levendowski D.J., Popovic D., Berka C., Westbrook P.R. (2012). Retrospective Cross-Validation of Automated Sleep Staging Using Electroocular Recording in Patients with and without Sleep Disordered Breathing. Int. Arch. Med..

[42] Popovic D., Khoo M., Westbrook P. (2014). Automatic Scoring of Sleep Stages and Cortical Arousals Using Two Electrodes on the Forehead: Validation in Healthy Adults. J. Sleep Res..

[43] Berry R.B., Brooks R., Gamaldo C., Harding S.M., Lloyd R.M., Quan S.F., Troester M.T., Vaughn B.V. (2017). AASM Scoring Manual Updates for 2017 (Version 2.4). J. Clin. Sleep Med..

[44] Gregersen N.T., Flint A., Bitz C., Blundell J.E., Raben A., Astrup A. (2008). Reproducibility and Power of Ad Libitum Energy Intake Assessed by Repeated Single Meals. Am. J. Clin. Nutr..

[45] Jakobsen J.C., Gluud C., Wetterslev J., Winkel P. (2017). When and How Should Multiple Imputation Be Used for Handling Missing Data in Randomised Clinical Trials–a Practical Guide with Flowcharts. BMC Med. Res. Methodol..

[46] Northeast R.C., Vyazovskiy V.V., Bechtold D.A. (2020). Eat, Sleep, Repeat: The Role of the Circadian System in Balancing Sleep–Wake Control with Metabolic Need. Curr. Opin. Physiol..

[47] Chaput J.-P. (2014). Sleep Patterns, Diet Quality and Energy Balance. Physiol. Behav..

[48] Zuraikat F.M., Wood R.A., Barragán R., St-Onge M.-P. (2021). Sleep and Diet: Mounting Evidence of a Cyclical Relationship. Annu. Rev. Nutr..

[49] VanItallie T.B. (2006). Sleep and Energy Balance: Interactive Homeostatic Systems. Metabolism.

[50] Saidi O., Rochette E., Bovet M., Merlin E., Duché P. (2020). Acute Intense Exercise Improves Sleep and Decreases next Morning Consumption of Energy-Dense Food in Adolescent Girls with Obesity and Evening Chronotype. Pediatr. Obes..

[51] Baker F.C., Lee K.A. (2022). Menstrual Cycle Effects on Sleep. Sleep Med. Clin..

