# Supplementary material for: The Effects of Different Modalities of an Acute Energy Deficit on Sleep and Next Morning Appetitive and Compensatory Behavior in Healthy Young Adults: The EDIES Protocol
Source: Nutrients. 2023 Apr 19;15(8):1962. doi: 10.3390/nu15081962 (PMC10144061; doi:10.3390/nu15081962)
Supplement: Supplementary file 1 [file nutrients-15-01962-s001.zip › nutrients-2294418-supplementary.pdf]

Table S1. Accelerometry analyses

Study period	Placement/algorithm	Methods	Outcomes
Run-in period (1 week)	Non-dominant wrist (Sadeh et al., 1994)	The Accelerometers (GT3X+, ActiGraph LLC, Pensacola, FL) will be used in conjunction with sleep logs.<br>Participants will be instructed to keep long sleeved clothing pulled above the monitor to allow ambient light exposure.<br>Wear-time validation will be performed on the data using vector magnitude. Periods with a zero value of vector magnitude for more than 5 minutes will be flagged as non-wear periods. In combination with the non-wear periods indicated on the subject sleep logs, true non-wear periods will be flagged on the actigraphy file. Furthermore, non-documented non-wear periods will be observed and documented.<br>Sleep analysis will be performed on each accelerometry file. Data will be then reintegrated into 60-second epochs for the analysis.<br>The beginning of the sleep episode will be first determined by a reduction in intensity and frequency of activity counts. The end of the sleep episode will be determined by an increase in intensity and frequency of activity counts.<br>If sleep logs and sleep period will be in concordance, sleep log will be recorded as beginning/end of sleep period. If not in concordance, activity counts will be used to designate sleep episode times.<br>The Sadeh algorithm will be used to identify sleep onset and offset. This algorithm was developed using an adult sample similar to our population (mean age of 22.6 yr). The Sadeh algorithm analyzes each epoch to determine if it should be designated as sleep or wake (using the five previous and the five future epochs).<br>After completing the sleep analysis an excel file will be exported containing sleep data, including total sleep time, total time in bed, bedtime, and wake up time.<br>All these data will be used to detect any deviation from the study protocol on both sleep/wake cycle and sleep duration during stabilization period. Participants not complying with the prescribed sleep schedule during the stabilization week (mean total time in bed < 420 min and / or mean bedtime delayed > 45 min) will be dropped from the analysis.	- TTB<br>- TST<br>- Bedtime<br>- Wake up time
Experimental sessions (24 hours)	Right hip (Freedson et al., 1998)	The Accelerometers (GT3X+), will be placed over participants' right hip on an elastic waistband at the anterior axillary line.<br>Data will be analyzed in 30-second epochs. Vector magnitude counts/min will be used to estimate active minutes (measurement of active minutes (MVPA); ≥3metabolic equivalents (METS).<br>Freedson et al. (2011) algorithm will be used to calculate AEE across the 24 hours wearing period. The Freedson 2011 equation to calculate AEE will be used.	- number of steps taken<br>- time spent in sedentary behavior<br>- time spent in light activity<br>- time spent in moderate PA<br>- Time spent in vigorous PA<br>- Time spent in MVPA<br>- AEE

TTB: total time in bed , TST: total sleep time, PA: physical activity, MVPA: moderate to vigorous physical activity, AEE: activity energy expenditure; All analyses will be performed via ActiLife 6 software (ActiGraph).

Table S2: Food items included in the standardized and *ad libitum* buffet meals

	Groups	Food	Nutritional values per 100g of food
		**Standardized meals**	
Breakfast	Dairy products	Bowl of milk	116 kcal
		Yogurt	78 kcal
	Juice	Orange	46 kcal
		Apple	49 kcal
	Grain (cereal)	Rusk	409 kcal
		Muesli	418 kcal
		Brioche	401 kcal
	Sweet product, sugars	Apricot jam	247 kcal
	Fats	Butter	753 kcal
Lunch	Grain (cereal)	Cooked rice	145 kcal
		Cooked pasta	126 kcal
		Crackers	450 kcal
	Seasoning (cooked)	Bolognese	227 kcal
		Pesto	370 kcal
	Dairy products	Yogurt	78 kcal
		Cottage cheese	311 kcal
		Grated cheese	390 kcal
	Sweet product, sugars	Applesauce	102 kcal
		Fruits in syrup	65 kcal
		Cake	458 kcal
Dinner	Main course (prepared food)	Ravioli (vegetal)	111 kcal
		Fish and vegetables	141 kcal
		Cantonese rice	164 kcal
	Grain (cereal)	Crackers	450 kcal
	Dairy products	Yogurt	78 kcal
		Cottage cheese	311 kcal
		Grated cheese	390 kcal
	Sweet product, sugars	Applesauce	102 kcal
		Fruits in syrup	65 kcal
		Cake	458 kcal
		***ad libitum* buffet**	
	Dairy products	Bowl of milk	116 kcal
		Yogurt	78 kcal
		Cheese (Emmental)	367 kcal

Juice	Orange	46 kcal
	Apple	49 kcal
Grain (cereal)	Rusk	409 kcal
	Muesli	418 kcal
	Brioche	401 kcal
Protein food	Egg	155 kcal
	White ham	125 kcal
Sweet product, sugars	Apricot jam	247 kcal
Fats	Butter	753 kcal
